# Emerging multifaceted roles of BAP1 complexes in biological processes

**DOI:** 10.1038/s41420-021-00406-2

**Published:** 2021-01-22

**Authors:** Aileen Patricia Szczepanski, Lu Wang

**Affiliations:** Simpson Querrey Center for Epigenetics and the Department of Biochemistry and Molecular Genetics, Northwestern University Feinberg School of Medicine, 303 East Superior Street, Chicago, IL 60611 USA

**Keywords:** Epigenetics, Biochemistry, Enzyme mechanisms

## Abstract

Histone H2AK119 mono-ubiquitination (H2AK119Ub) is a relatively abundant histone modification, mainly catalyzed by the Polycomb Repressive Complex 1 (PRC1) to regulate Polycomb-mediated transcriptional repression of downstream target genes. Consequently, H2AK119Ub can also be dynamically reversed by the BAP1 complex, an evolutionarily conserved multiprotein complex that functions as a general transcriptional activator. In previous studies, it has been reported that the BAP1 complex consists of important biological roles in development, metabolism, and cancer. However, identifying the BAP1 complex’s regulatory mechanisms remains to be elucidated due to its various complex forms and its ability to target non-histone substrates. In this review, we will summarize recent findings that have contributed to the diverse functional role of the BAP1 complex and further discuss the potential in targeting BAP1 for therapeutic use.

## Facts

- The BAP1 complex is an evolutionary conserved, multiprotein complex that functions as a general transcriptional activator via deubiquitination of H2AK119Ub and other epigenetic/transcription factors.
- Variations within the composition of subunits encompassing these BAP1 complexes can determine context-specific or tissue-specific functions. Being universally expressed, the BAP1 complex and its associated subunits play a critical and fundamental role in maintaining development, metabolic processes, and tumorigenesis.
- Both loss-of-function and gain-of-function mutations within these BAP1 complexes have been identified in both developmental diseases and cancers, indicating the need for fine-tune adjustments in the BAP1 complex machinery in order to sustain appropriate levels for catalytic activity and accurate cellular localization, which are critical for the determinant of cell fate and transformation. The BAP1 complex has emerged as an ideal therapeutic target for treatments involving developmental/metabolic diseases and human cancers.

## Open questions

- What is the mechanism behind the recruitment of the BAP1 complex to chromatin, and which factors determine its chromatin binding specificity?
- What is the crystal structure of the human BAP1 complex?
- What are additional factors associated with functional compositions of these BAP1 complexes in both nuclei and cytosol? Does BAP1 require other subunits to be functional in the cytosol vs. nuclei?
- What are the function and tissue specificity between different compositions of these BAP1 complexes?
- Do all the BAP1 substrates contain similar or identical BAP1-binding motifs, and/or conserved lysine residues?

## Compositions of the BAP1 complex

Ubiquitin (Ub) is a highly conserved, stable, and ubiquitously expressed protein found in all tissues and eukaryotic organisms^1,2^. The Ub system is significant due to its central role in the maintenance of nearly all cellular processes. However, one way to regulate Ub activity is within the Ub-dependent proteasome pathway, which includes enzymes such as deubiquitinases (DUBs) that function to catalyze the irreversible conjugation process of Ub or Ub-like proteins from substrates^3^. The human genome encodes approximately 100 DUB enzymes belonging to seven different families, which exhibit distinct but overlapping cleavage preferences^4^—such as the ubiquitin C-terminal hydrolase (UCH) subclass, which consists of four members (UCHL1, UCHL3, UCHL5, and BAP1) that share close homology in their catalytic domain^5^. Here on, we will focus on BAP1’s functional role in its complex form.

**Fig. 1:**
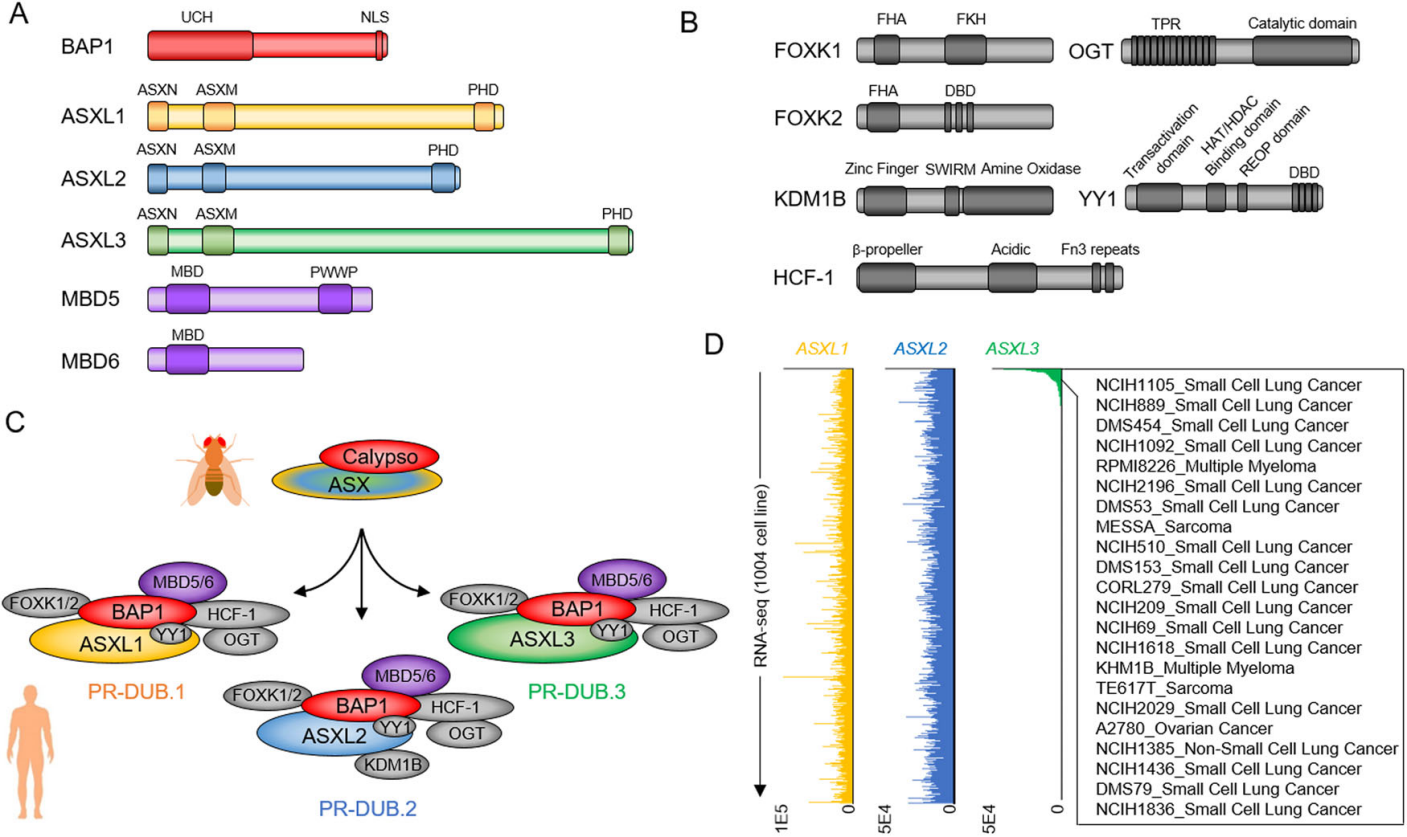
Compositions of the Polycomb repressive deubiquitinase PR-DUB (BAP1) complex. **A** Domain organization of human BAP1, ASXL1, ASXL2, ASXL3, MBD5, and MBD6 proteins. **B** Domain organization of human FOXK1, FOXK2, KDM1B, HCF-1, OGT, and YY1 proteins. **C** Compositions of the Drosophila Calypso/ASX complex and human PR-DUB.1, PR-DUB.2, and PR-DUB.3 BAP1 complexes. **D** The counts per million (CPM) value for ASXL1/2/3 expression in 1,004 human cell lines.

Initially, BAP1 was identified as a BRCA1-associated protein that interacts with the BRCA1-RING finger domain^6^. Further analysis indicated that BAP1 is a novel 729 amino acid-length protein that consisted of a functional domain within 240 amino acids of the amino-terminal, which showed significant homology to other known thiol proteases of the UCH family (Fig. 1A)^7^. Evolutionarily speaking, BAP1 has conserved functional origins going as far back into invertebrate models, such as Drosophila^8^. In Drosophila, a previously uncharacterized gene, known as *Calypso*, encoded for a 471 amino acids long, polypeptide chain—which was found to be closely related to the BAP1 human homolog. Through affinity purification methods, Calypso was then found to be co-purified with a PcG protein, known as Additional sex combs (ASX) by Müller’s lab^8^ (Fig. 1B). The Calypso/ASX complex was then further demonstrated to function as a Polycomb Repressive-Deubiquitinase (PR-DUB) of Drosophila histone H2AK118 via its binding at the Polycomb response elements (PREs) of PcG targeted genes in Drosophila^8^. Consequently, mutations in Calypso that disrupt H2A deubiquitinase activity impair the repression of Hox genes in Drosophila, suggesting a critical function for the Calypso/ASX complex in transcriptional regulation and development^8^.

In human cells, further advancements were made by a cluster of studies that have identified subunits associated with transcriptional regulatory functions that can form complexes with BAP1, such as the transcriptional co-regulator host cell factor-1 (HCF-1)^9,10^, the transcription factor Yin Yang 1 (YY1)^11^, and the multifaceted transcription factor forkhead box proteins K1/2 (FOXK1/2)^12^ (Fig. 1B). From a different study, these findings were expanded upon through methods involving purification of endogenous BAP1, which showed that BAP1 had stable protein–protein interactions with HCF-1, along with four epigenetic-related subunits: *O*-linked- N-acetylglucosamine transferase (OGT), lysine-specific histone demethylase 1B (KDM1B or LSD2), and putative PcG proteins called additional sex combs-like 1/2 (ASXL1/2)^13^. In subsequent studies, two members of the methyl-CpG-binding domain family (MBD5/6)^14^ and ASXL3^15,16^ were also shown to interact with BAP1 (Fig. 1B). However, MBD5/6 can only be pulled down by a subset of components within the BAP1 complex (BAP1, ASXL2, and KDM1B) in vitro via its MBD domain. Thus, the interactions between mammalian BAP1 with MBD5/6 would need further validation with future studies.

Based on these previous findings, the human BAP1 complex is a multiprotein complex that contains as many as ten different subunits. Although several subunits—such as FOXK1/2, KDM1B, YY1, HCF-1, and OGT—have also been characterized as components within other protein complexes (Fig. 1A, B). Interestingly, only a few subunits (e.g., ASXL1-3) are exclusive within these BAP1 complexes^14^. Collectively, there should be several versions of these BAP1 complexes existing within mammalian cells^17,18^. The three ASXL subunits within each designated BAP1 complexes are shown to be conserved from Drosophila to human (Fig. 1C). Interestingly, different from ASXL1 and ASXL2, which are both expressed at a bulk level across most of the cell lines, the expression of ASXL3 is more tissue-specific and shown to be strongly enriched in neuroendocrine cell lines such as human small cell lung cancer cells (Fig. 1D)^16^. This result suggests that there is a distinctive function in the chromatin localization among BAP1–ASXL1/ASXL2/ASXL3 complexes.

## The functions of the BAP1 complex at the chromatin level

**Fig. 2:**
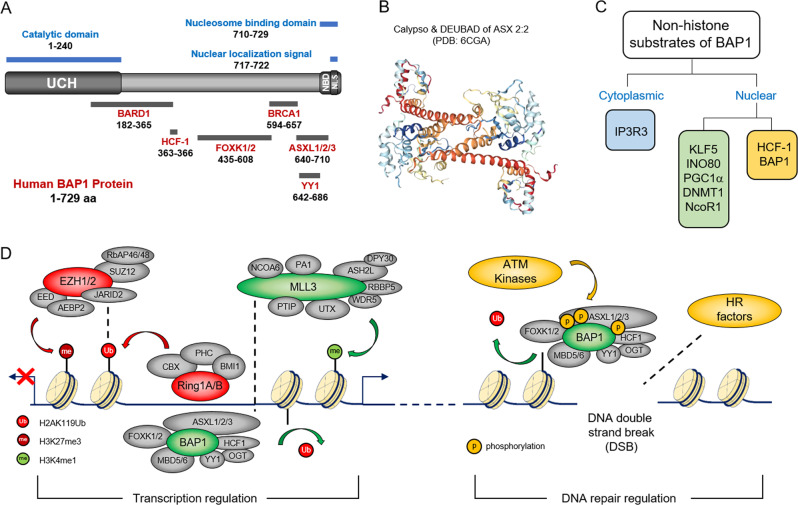
BAP1 complex’s function at chromatin. **A** Domain organization of human BAP1 and the protein–protein interaction interface between BAP1 and other factors. **B** The crystal structure of Calypso/DEUBAD of ASX dimer 2:2 complex, modified from PDB: 6CGA^22^. **C** List of known BAP1 substrates. **D** The transcriptional regulation machinery comprised of the PRC1 complex, PRC2 complex, BAP1 complex, and MLL3 COMPASS, and the DNA double-strand break repair machinery comprised of ATM, the BAP1 complex, and homologous recombination factors.

As a member of the UCH family, BAP1 protein is comprised of a conserved N-terminal catalytic domain (UCH) (1–240 aa), as well as a homology C-terminal UCHL5/UCH37-like domain (ULD) (640–710 aa) (Fig. 2A). The C-terminus of BAP1 is most likely important for the assembly and stability of the BAP1 complex, due to its protein–protein interaction interfaces with BRCA1^6^, ASXL1-3^19^, and YY1^11^ (Fig. 2A). Furthermore, there are overlapping domains at the C-terminal extension of BAP1, which consists of a putative nuclear localization signal (NLS) sequence and a positively charged nucleosome binding domain (NBD), that auto-recruits BAP1 to substrate nucleosomes^19^ (Fig. 2A). Besides these two relatively conserved, functional domains within BAP1 N-terminus and C-terminus regions, BAP1 contains an additional ~395 amino acids. This middle proteomic region is considered to be a unique linker between UCH and C-terminus domains by providing another interface between BAP1 and other factors, including BARD1^20^, FOXK1/2^12^, and HCF-1^10^ (Fig. 2A). Previously, it was shown that the E2/E3 hybrid ubiquitin ligase UBE2O directly modifies BAP1 NLS, and thus depletion of UBE2O impairs proper nuclear localization of BAP1^21^. However, details pertaining to the crosstalk and molecular mechanisms between overlapping domains NLS and NBD remains unknown.

To fully understand how the BAP1 complex assembles and functions, two independent groups contributed towards the development of Drosophila Calypso/ASX crystal structures, providing details that could relate to the collaboration between mammalian ASXL proteins and BAP1 (Fig. 2B). Based on the structure published by Mace’s group, they generated a model of Calypso and its activating partner ASX forming a bidentate 2:2 complex, where two Calypso/ASX heterodimers dimerize via the Calypso coiled-coil regions^22^. However, in the crystal structure published by Müller’s group, they did not observe the 2:2 complex under their experimental conditions. Although, they did observe a dimer of heterodimers at the same interface of coiled-coil regions, but only within the asymmetric unit of their crystal structures. Therefore, the structure of a Calypso/ASX nucleosome complex has been suggested in order to clarify whether or not dimerization exists^23^. However, within human cells, two different groups have identified a similar complex via mass spectrometry analysis consisting of a 2:1 stoichiometry for the BAP1/ASXL1 complex^14,19^. Intriguingly, this may indicate for a more complex structure and/or potential regulatory mechanism involving the human BAP1 complex in vivo.

BAP1 is identified as a major deubiquitinase of H2AK119Ub among all of the ~100 DUBs via a targeted, small-scale shRNA screening^24^. Recent CRISPR screening studies by Dixit’s group further identified that depletion of PRC1 subunit Ring1B (but not Ring1A) could rescue the cell death induced by loss of BAP1^25^, indicating that there is a robust and direct epigenetic dynamic occurring between the PRC1 and BAP1 complexes that require a balanced state to properly regulate gene expression and determine cell fate. Additional studies have also demonstrated that H2AK119Ub could be targeted by the binding site of the UIM domain within subunit JARID2, which can provide a stronger affinity in order to recruit the PRC2 complex and inactivate transcription (Fig. 2C)^26–28^. In mouse models, depletion of BAP1 results in an increase of H3K27me3 levels, and repression of PRC2 targets^29^. Consistent with these previous findings, depletion of BAP1 by CRISPR in human cell lines also leads to a significant increase of H3K37me3 levels^30^, primarily due to the loss of MLL3 COMPASS (complex of proteins associated with Set1) recruitment to BAP1-dependent enhancers. Overall, these results established an epigenetic/transcriptional balance model between PRC1/PRC2, BAP1, and COMPASS complexes, representing a mechanism of “switching on/off” targeted enhancer activity and gene expression^31^ (Fig. 2C). Interestingly, during the cellular response to DNA double-strand breaks (DSBs) induced by UV irradiation or DNA damaging agents^32^, BAP1 could be phosphorylated by DDR kinase ATM, and then recruited to the chromatin near DSB sites^32,33^. It was further demonstrated that both of the catalytic activity and the phosphorylation of BAP1 by ATM is required for efficient assembly of the homologous recombination (HR) factors BRCA1 and RAD51 at the DSB loci (Fig. 2C)^34^. Thus, how BAP1 was recruited to the DSB site, and determining which subunit within these BAP1 complexes can mediate this function may need to be further investigated.

Besides the removal of mono-ubiquitin from histone H2AK119Ub, BAP1 can also cleave more massive Ub derivatives due to its relatively longer active-site crossover loop (usually >14 residues) compared to other UCHs—such as single-domain enzymes UCHL1/3^35^ (Fig. 2B). Therefore, the length of the active-site crossover loop of UCHs determines catalytic activity and substrate specificity of ubiquitin chains and thus provides BAP1 with the potential to hydrolyze a wide range of Ub derivatives, including isopeptide Ub chains of K48-diUb^36^ and other non-histone substrates. For instance, one of the subunits within the BAP1 complex, HCF-1, demonstrated being the first non-histone substrate of BAP1 (Fig. 2D). BAP1 was shown to deubiquitinate HCF-1 at the N-terminus, which is essential for BAP1 binding and BAP1-dependent cell growth inhibition^10^. Other epigenetic/transcription factors such as Krueppel-like factor 5 (KLF5)^37^, chromatin-remodeling ATPase INO80^38^, DNA methyltransferase 1 (DNMT1)^39^, nuclear receptor corepressor-1 (NCoR1)^40^, peroxisome proliferator-activated receptor gamma coactivator 1-alpha (PGC1-α)^41^, and type 3 inositol-1,4,5-trisphosphate receptor (IP3R3)^42^ are all identified as BAP1 unique non-histone substrates (Fig. 2D). Although a conserved deubiquitination site has yet to be identified among these non-histone substrates, most of them are shown to be stabilized via deubiquitination catalyzed by BAP1.

## BAP1 complex’s function in metabolism

**Fig. 3:**
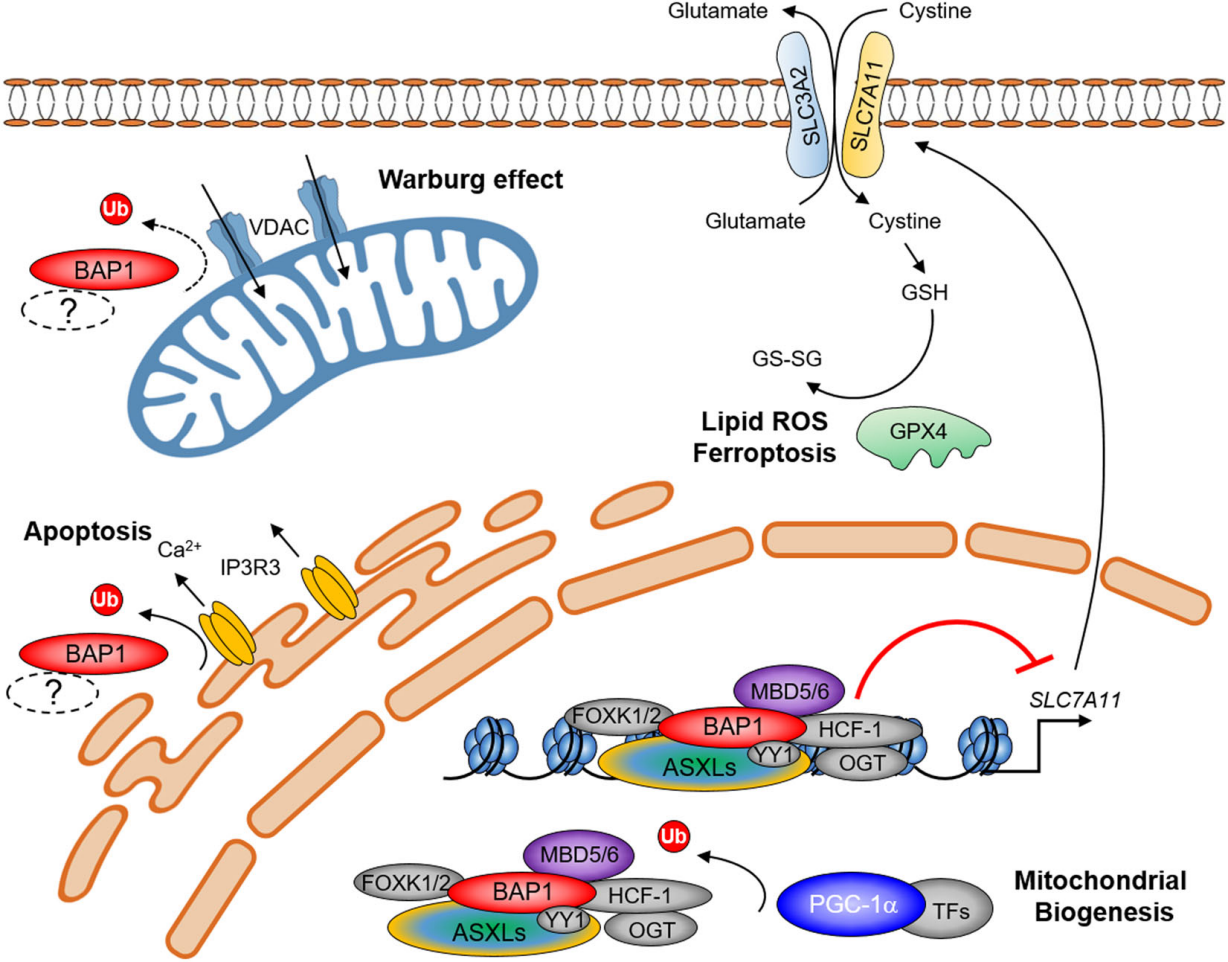
BAP1 complex’s function in metabolism. In nuclei, BAP1 occupies the promoter region of *SLC7A11* gene, and negatively regulates the expression of SLC7A11, which mediates the uptake of extracellular cystine. Loss of BAP1 activates the glutathione biosynthesis, which is utilized by GPX4 to detoxify lipid hydroperoxide and to protect tumor cells from ferroptosis. BAP1 could also function as a deubiquitinase for non-histone substrate PGC-1α, which protects PGC-1α from degradation and promotes gluconeogenesis. In the cytosol, BAP1 is critical for the stability of IP3R3 via deubiquitination, which mediates Ca^2+^ flux to mitochondria by suppressing cell transformation (as well as gatekeeper VDAC) and allowing passage of metabolites, nucleotides, and ions in/out of the mitochondria.

The epigenetic balance between PRC1/PRC2, BAP1, and COMPASS complexes determines whether gene expression will be “switched on/off”, indicating that BAP1 may play an important role in regulating multiple functions within different tissue types, as well as diverse cellular processes through epigenetic mechanisms (Fig. 2C). BAP1 is a ubiquitously expressed deubiquitinase; however, its potential roles are just beginning to be established in different biological processes. For instance, studies have currently linked the BAP1 complex to the maintenance of metabolic homeostasis. In addition, emerging evidence from various research groups indicated that BAP1 might have significant metabolic roles impacting other biological components while being localized within both the cytosol and nucleus (Fig. 3).

In 2012, Yang’s group has identified that HCF-1, a known component of the BAP1 complex, recruits subunit OGT to O-GlcNAcylate PGC1α, and therefore O-GlcNAcylation would facilitate the binding of deubiquitinase BAP1 in order to protect PGC1α from degradation and be able to promote the metabolic pathway of gluconeogenesis. Dysregulation of gluconeogenesis is a major cause of hyperglycemia among diabetic patients, and the OGT/HCF-1/BAP1 protein complex may be a potential therapeutic target in order to improve glucose homeostasis within diabetic mice^41^ (Fig. 3).

More recently, the nuclear BAP1 protein complex was shown to also play an essential role in negatively regulating the expression of cystine transporter solute carrier family 7 member 11 (*SLC7A11*), which makes up the cystine/glutamate xCT amino acid antiporter and antagonizes glutamate metabolism in cells. Typically, BAP1 activates gene expression however in this particular case BAP1 represses *SLC7A11* gene expression in a deubiquitinating-dependent manner by removing H2AUb from the *SLC7A11* promoter. Furthermore, since BAP1 inhibits cystine uptake by repressing SLC7A11 expression, it prevents glutathione (GSH) biosynthesis, leading to an increase in lipid reactive oxygen species (ROS), which promotes ferroptosis and tumor suppression^43^ (Fig. 3). However, the underlying mechanism of BAP1’s catalytic activity-mediated transcriptional repression remains largely unexplored. It would be interesting to test whether if the transcriptional repression effect and/or H2AUb dynamics induced by loss of BAP1 could be rescued by PRC1 inhibition.

In addition, Dey’s group took the advantage of developing a model involving neutron-encoded (NeuCode) lysine isotope labeling of mice, as a strategy for multiplexed proteomic analysis in BAP1-depleted mice. In their study, they observed that BAP1 deletion in the liver can potentially cause mitochondrial stress due to an increase in ubiquitination of outer mitochondrial membrane proteins, such as VDAC1/2/3 and Maob, suggesting a potential function of BAP1 in the cytosol. Moreover, the pancreas showed reduced expression of mitochondrial proteins^44^. In consistent with previous results, it has been shown that normal primary cells carrying heterozygous germline *BAP1* mutations in humans have been shown to increase aerobic glycolysis (also known as the “Warburg effect”) and impair mitochondrial respiration/ATP production. Therefore, these results suggest that cells with BAP1 loss-of-function mutations may rely heavily on the glycolytic metabolic pathway for energy production, eventually leading to the development of one and often several malignancies^45^ (Fig. 3).

Initially, it was Carbone’s group that provided evidence of BAP1 cytosol localization in patient-derived fibroblast cells. The results have shown that BAP1 was clearly detected as being localized at the endoplasmic reticulum (ER) by electron microscopy and immunofluorescence. In this case, BAP1 was identified as being able to bind, deubiquitylate, and stabilize type 3 inositol 1, 4, 5-trisphosphate receptor (IP3R3), which modulates calcium (Ca^2+^) release from the ER into the cytosol and mitochondria, promoting apoptosis. Reduced levels of BAP1 in heterozygous carriers results in a reduction of both IP3R3 and Ca^2+^ flux, preventing accumulated DNA damage cells from undergoing apoptosis^42^ (Fig. 3). Interestingly, based on the findings from a recent work published by the Margueron group, there is no detectable BAP1 protein in ASXL1/ASXL2 double knockout cells, whereas BAP1’s transcript levels were unaffected^46^. This result suggests that BAP1 may only function within a complex with either ASXL1 and/or ASXL2 in the cytosol. However, purification of BAP1 from both nuclei and cytoplasm will be required to fully address this question.

Based on previous studies by El Bachir Affar’s group, the ubiquitin-conjugating enzyme UBE2O could promote cytoplasmic localization of BAP1^21^. It will be interesting to purify BAP1 from the cytoplasm in the absence of UBE2O. Overall, these studies have shown strong supportive data that links BAP1’s functional role in several metabolic pathways, and how dysfunction can promote other pathological issues, including the development of cancer.

## BAP1 complex’s function in the development

Originally, PR-DUB BAP1 (Calypso) was co-purified in existence with its activating Polycomb group (PcG) protein, Additional sex combs (ASX)^8^, which is required for both repressive and active transcriptional states of homeotic loci^47^. Interestingly, an ASX mutant allele was identified to cause both anterior (regulated via PcG repressive genes) and posterior (regulated via Trithorax group (TrxG) activating genes) homeotic transformations in Drosophila. Thus, it is likely that BAP1 has an important function in embryonic development since it relies heavily on its association with ASX to protect transcriptionally active developmental genes against silencing from PRC1 ubiquitination. In *Xenopus laevis* development, BAP1 loss leads to transcriptional silencing of key genes regulating pluripotency-to-commitment transition into ectoderm, mesoderm, and neural crest lineages, due to lack of H3K27Ac levels accumulating at those genes loci. In mammals, the first BAP1 knockout (KO) phenotype was created in mice embryos from Dixit’s group^13^. They observed that *BAP1* gene deletion leads to embryonic lethality due to developmental retardation between E8.5 and E9.5 days, indicating a critical role for BAP1 during embryogenesis (Table 1).

**Table 1:** BAP1 complex in development.

Subunit	Phenotype in KO mice	Linked to human developmental diseases	References
BAP1	Developmental retardation and embryonic lethality (E8.5-E9.5 days)	**–**	^13^
ASXL1	Embryonic lethality (>E18.5 days), dwarfism, anophthalmia, microcephaly, kidney podocyte defects, and craniofacial defects	Mutations were discovered in patients with Bohring–Opitz syndrome that have a segmental overgrowth	^48,75–77^
ASXL2	>50% pups die before birth, 21.7% pups die within two months, axial skeletal abnormalities, enlarged heart, and reduced bone mineral density (BMD)	**–**	^78,79^
ASXL3	**–**	Mutations were discovered in patients with Bainbridge–Ropers syndrome	^15,80^
MBD5	Abnormal social behavior, cognitive impairment, and motor/craniofacial abnormalities.	Mutations were discovered in patients with intellectual disability, epilepsy, and developmental delay	^54,81–83^
MBD6	**–**	Mutations were discovered in patients with an autism spectrum disorder	^84^

As core subunits within BAP1 complexes, all three of the ASXL proteins (ASXL1-3) were found to be essential in different developmental processes. In ASXL1 conditional-KO mice ES cells, constitutive germline loss of *Asxl1* also resulted in embryonic lethality (similar to *BAP1* depletion) as well as craniofacial abnormalities. Although some of the embryos were detected at E18.5 days, all of the pups died before birth^48^ (Table 1). Depletion of ASXL1 in mice could also lead to developmental abnormalities, including anophthalmia, microcephaly, cleft palates, and mandibular malformations, which are all common features associated with Bohring–Opitz syndrome among human patients. Furthermore, ASXL1 loss-of-function mutations were also observed by whole-exome sequencing in patients with Bohring–Opitz syndrome^49^. Recently, a de novo truncating mutation in ASXL1 was detected in an individual with abnormalities, including severe hypotonia, developmental delay, a mid-line capillary malformation, and distinctive craniofacial features^50^.

Comparing with ASXL1-KO mice, 50% of ASXL2-KO mice will die before birth, and around a quarter of the pups die within two months after birth (Table 1). In addition, ASXL2-KO mice survivors had unique phenotypes distinctive from ASXL1-KO mice, including axial skeletal abnormalities, enlarged hearts, and some of them even have reduced bone mineral density^51^. These animal experiments have established separate functional roles between ASXL1 and ASXL2, in which neither of them could compensate for the function of one over the other. To date, there are no documented reports on the generation or phenotypes of ASXL3-KO mice. However, ASXL3 mutations were observed in some human neurological diseases, such as Bainbridge–Ropers syndrome and autism spectrum disorder (ASD)^15,52^. In a recent publication using the *Xenopus laevis* genetic model, it was demonstrated that loss of ASXL3 protein during early embryo development highly perturbs neural cell fate specification, potentially resembling the Bainbridge–Ropers syndrome phenotype in humans^53^. These results from patients suggested a critical function of ASXL3 in neuronal development, differentiation, and function. Indeed, in our recent studies, we demonstrated that in comparison to ASXL1/2, ASXL3 is a more tissue-specific additional sex combs-like protein that is essential for BRD4-dependent enhancer activation in neuroendocrine cancer^16^.

Furthermore, BAP1 complex-specific associated proteins, MBD5/6, were also found to be involved in neurologically-related diseases. For instance, MBD5/6 mutations were found in patients with intellectual disability, epilepsy, and developmental delay^54^. Consistent with phenotypes observed in human patients, MBD5-deficient mice also exhibited abnormal social behaviors, cognitive impairment, and motor/craniofacial abnormalities^55^. Currently, MBD5/6 are known heterochromatin-binding factors. However, determining whether if MBD5/6 contributes to the BAP1 complex’s function explicitly at the chromatin, or if it partakes in a recruitment mechanism to heterochromatin remains mostly obscure.

## BAP1 complex in cancer: friend or foe?

**Fig. 4:**
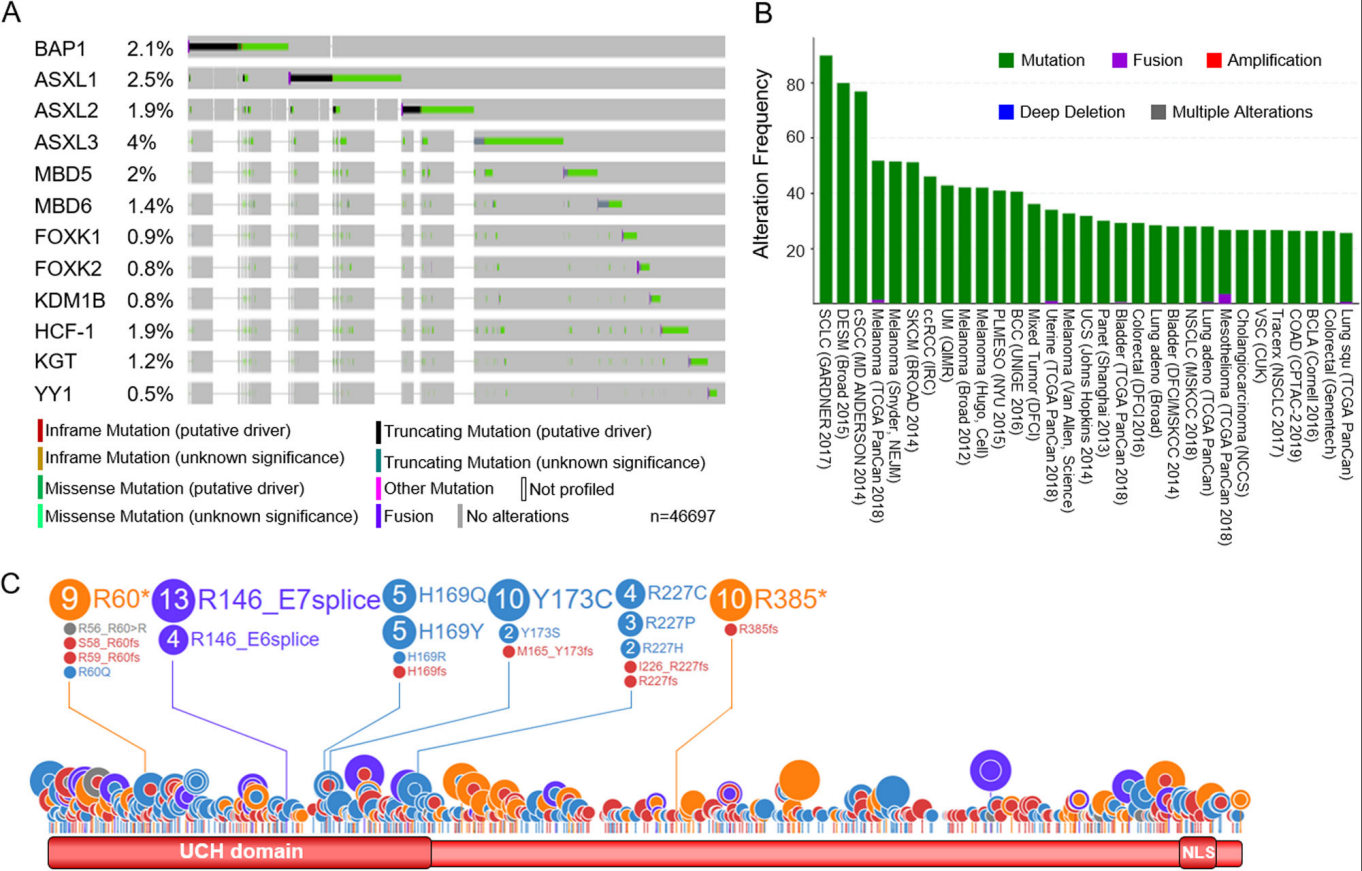
BAP1 complex mutations in cancer. **A** General mutation status of BAP1 and the subunits of BAP1 complex in pan human cancers by cBioPortal database (*n* = 46697) (https://www.cbioportal.org/). **B** Mutation frequencies of subunits within the BAP1 complex across various human cancer types as obtained from cBioPortal. **C** Distribution of all BAP1 mutations that occur within the BAP1 protein-coding region as obtained from St. Jude Cloud PeCan (https://pecan.stjude.cloud/proteinpaint/).

Recent studies have shown that the dysregulation of epigenetic enzymes plays a crucial role in tumorigenesis^56–59^. Both loss-of-function and gain-of-function mutations within epigenetic factors were observed in many types of human cancers^60,61^. BAP1 complex, which contains as many as ten different subunits (Fig. 1), is one of the most highly mutated epigenetic complexes among human cancers^62–65^. Different types of mutations, especially missense and truncating mutations, were observed in most of the subunits within the BAP1 complex (Fig. 4A) across different cancer types (Fig. 4B).

In BAP1, most of the somatic mutations were detected in the N-terminal UCH domain, and are identified as catalytic inactive mutations^66^. Interestingly, in comparison to somatic mutations, most of the germline mutation hotspots within BAP1 were located near the CTD^67^ (Fig. 4C). As a result, germline mutations may affect the NLS within BAP1’s CTD^21^, which would lead to an accumulation of BAP1 in the cytosol and may then further disrupt proper metabolic processes (Fig. 3B). Moreover, germline mutations in *BAP1* have also been associated with *BAP1*-tumor predisposition syndrome that consists of multiple tumors, such as the following in descending order of frequency: uveal melanoma (UM), malignant mesothelioma (MMe), cutaneous melanoma (CM), clear cell renal cell carcinoma (ccRCC), and basal cell carcinoma (BCC)^68,69^.

To determine the potential tumor-suppressive function of BAP1 in mammals, Dixit’s group has generated the first BAP1 conditional knockout (cKO) mice. Based on their findings, BAP1-cKO mice leads to the development of splenomegaly within 4 weeks of BAP1 depletion, and myeloid cells were significantly increased in lymph nodes and bone marrow in BAP1-cKO mice, resulting in myeloid transformation^13^. Mechanistically, Levine’s group has further demonstrated that BAP1 loss in mice resulted in increased levels of H3K27me3 due to an elevated expression of EZH2, which leads to enhanced repression of Polycomb targeted genes^29^. Thus, BAP1-null tumor cells are more sensitive to EZH2 inhibitor treatment in vivo. In addition, our previous genome-wide studies have shown that enhancers binding to the BAP1 complex are responsible for the recruitment of MLL3 COMPASS, which functions as a general tumor suppressor (Fig. 2C). Depletion of BAP1 leads to a dramatic decrease of MLL3 COMPASS and its subunit, H3K27 demethylase UTX, leading to further repression of multiple tumor suppressors, such as GRHL2, RBMS3, DACT2, and DSC3^30^. In melanoma, the constitutively active, oncogenic form of BRAF (BRAF^V600E^) combined with BAP1 loss was seen in 67% of BAP1 tumor syndrome-associated lesions^70^. Consequently, BAP1 loss cooperates with BRAF^V600E^ to increase susceptibility to DNA damage, promote tumor growth, and metastasis both in vitro and in vivo^71^.

Although genetic depletion of BAP1 could lead to leukemogenesis in vivo, emerging studies have identified that BAP1 may also be required for tumor cell viability. In human breast cancer, BAP1 functions as deubiquitinase to stabilize the transcription factor KLF5—which is highly expressed in basal-like breast cancer—and promotes breast cancer cell proliferation, migration, and tumor growth. Furthermore, depletion of BAP1 in breast cancer cell line HCC1806 phenocopies KLF5 depletion and significantly reduced tumor growth in vivo^37^. In head and neck squamous cell cancer (HNSCC), BAP1-mediated histone H2AK119 mono-ubiquitination was found to be involved in radioresistance. The expression levels of BAP1 was significantly associated with poor prognosis in patients with HNSCC. This study reveals that BAP1 may be a potential therapeutic target in HNSCC clinical treatment^72^. In fact, based on a recent study by Yang’s group, a proper level of BAP1 expression/activity is critical for the maintenance of cell fate. In terms of leukemogenesis, hyperactivation of BAP1 was observed in ASXL1 truncated leukemia cells. In addition, BAP1 hemizygous deletion in ASXL1^Y588X^ Tg mice—which have impaired hematopoietic stem/progenitor cell function and diverse myeloid malignancies—is sufficient to prevent mutant ASXL1-driven based myeloid differentiation and myeloid malignancy^73^. Collectively, these studies have shown the dichotomic functional role of BAP1 in different cancer types, as a tumor suppressor and/or tumor promoter, which further supports the notion of BAP1’s complexity.

## Future directions

To date, the BAP1 complex was discovered for over two decades now and has been actively investigated throughout the years, with emerging studies unfolding its complex nature^74^. As a general transcriptional activator and a major deubiquitinase of histone H2AK119, the BAP1 complex establishes an epigenetic balance with the PRC1 complex to determine the “switching on/off” of transcriptional activity, ultimately affecting targeted gene expression. Besides histone H2AK119, a remarkable group of non-histone substrates of BAP1 has also been identified, which all play a critical role in a variety of biological processes, including metabolism and development. Dysregulation or mutations in the BAP1 complex was demonstrated to be critical during tumorigenesis. Due to the number of possible conformations and the functional complexity of this multiprotein complex, the BAP1 complex regulation and the factor that determines its chromatin binding or cytoplasmic localization remains unclear. In fact, loss-of-function and gain-of-function mutations in the BAP1 complex have both been recognized as potential drivers in different disease models, especially cancers^65,69^. Nevertheless, it seems that the proper activity and localization of BAP1 is essential for the determinant of cell fate. Thus, the development of small-molecule modulators of BAP1 will be required for future therapeutic studies to further investigate its complexity and be able to potentially develop effective clinical treatments.
